# Positioning of proteasome inhibitors in therapy of solid malignancies

**DOI:** 10.1007/s00280-017-3489-0

**Published:** 2017-11-28

**Authors:** Margot S. F. Roeten, Jacqueline Cloos, Gerrit Jansen

**Affiliations:** 1 Department of Hematology, VU University Medical Center, Amsterdam, The Netherlands; 2 Department of Pediatric Oncology/Hematology, VU University Medical Center, Amsterdam, The Netherlands; 3 Amsterdam Rheumatology and Immunology Center, Location VUmc, VU University Medical Center, Amsterdam, The Netherlands

**Keywords:** Proteasome inhibitors, Bortezomib, Carfilzomib, Solid tumors, Drug resistance

## Abstract

Targeting of the protein degradation pathway, in particular, the ubiquitin-proteasome system, has emerged as an attractive novel cancer chemotherapeutic modality. Although proteasome inhibitors have been most successfully applied in the treatment of hematological malignancies, they also received continuing interest for the treatment of solid tumors. In this review, we summarize the current positioning of proteasome inhibitors in the treatment of common solid malignancies (e.g., lung, colon, pancreas, breast, and head and neck cancer), addressing topics of their mechanism(s) of action, predictive factors and molecular mechanisms of resistance.

## Introduction

For many types of cancer, chemotherapy is the first choice of treatment. However, some cancers are intrinsically resistant (refractoriness), while others develop resistance during treatment (acquired resistance) [1]. The recurrent problem of drug resistance urges the discovery of new drugs with novel mechanisms of action. Over the past decade, several classes of drugs have been developed that specifically target the process of proteasomal protein degradation via the ubiquitin-proteasome system (UPS) [2]. Proteasome activity is essential for cell homeostasis and is also controlling (by ubiquitination of key proteins) various selected process in cancer cells, e.g., cell cycle control (cyclins, cdk inhibitors), oncogenic transformation (N-myc, c-jun), tumor suppression (p53), apoptosis (Bax) and regulation of transcription factors (NFκB) [2–4]. From this perspective, it is not unexpected that proteasome inhibitors (PIs) have shown promising anti-cancer efficacy.

Bortezomib (BTZ) represents a first-generation PI being approved by the Food and Drug Administration (FDA) and European Medicines Agency (EMA) for the treatment of multiple myeloma (MM) and mantle cell lymphoma (MCL) [5]. Nowadays, BTZ is used as a front-line therapy for MM and in combination with autologous stem cell transplantation, the survival of patients with MM compared to conventional therapy has doubled [6]. In other hematological malignancies, e.g., acute leukemia, PIs also showed promising results [7]. However, BTZ faces several limiting factors impacting its short and long-term success, such as toxicity related to off-target effects and acquisition of resistance [8–12]. To this end, next generation PIs were developed to overcome some of these limiting factors.

Despite the success of PIs in the treatment of hematological malignancies, in solid tumors, the clinical efficacy of BTZ as a single agent is limited [13]. Second-generation PIs might have more effect on solid tumors, due to different selectivities and inhibitory potencies for proteasome subunits, along with reduced side effects. The aim of this review is to summarize the current positioning of PIs in the (combination chemotherapy) treatment of common solid malignancies, addressing topics of mechanisms of action, predictive factors and molecular mechanisms of resistance.

## The proteasome

### Structure of the 26S proteasome and enzymatic activities

**Fig. 1:**
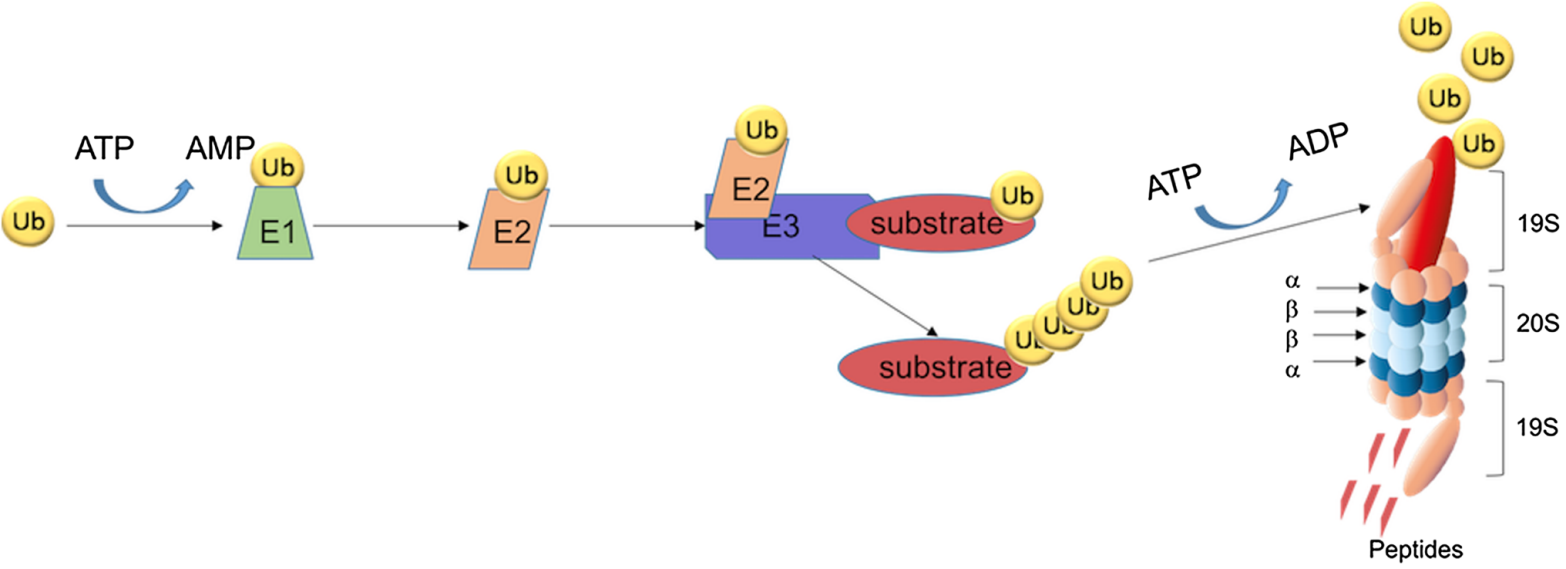
The UPS-system. Ubiquitin (Ub) is activated by the activating enzyme E1, Ub is then transferred to the conjugating enzyme E2. The ligase E3 enzyme attaches Ub to the target protein (substrate) and a substrate with at least four Ub moieties is then recognized by the proteasome for degradation. The 19S cap of the proteasome removes the Ub moieties after which the substrate is degraded in the 20S into smaller peptides

The proteasome is part of the UPS which is crucial for the intracellular homeostasis and responsible for degrading 80–90% of the intracellular proteins [3, 14, 15]. In normal cells, regular protein degradation imposes a large burden for the UPS and the balance of synthesis and degradation is tightly regulated. The UPS controls this balance by tagging damaged or misfolded proteins with multiple ubiquitin moieties serving as a signal for degradation by the proteasome. The process of protein (poly)ubiquitination involves a cascade of 3 enzymatic steps; ubiquitin-activating enzymes (E1), ubiquitin-conjugating enzymes (E2) and ubiquitin E3 ligases (Fig. 1). For protein degradation, the target protein must be linked with four or more ubiquitin units [16]. Prior to actual degradation by the proteasome, deubiquitinases (DUBs) remove and recycle ubiquitin moieties from the tagged proteins [17, 18].

The constitutive 26S proteasome consists of the 20S catalytic core domain and two 19S regulatory particles. The 19S subunits bind to both ends of the 20S core proteasome and is responsible for the recognition of the polyubiquitinated proteins, facilitating the removal of the polyubiquitin chains, unfolding the protein and skewing into the 20S core [19, 20]. The 20S catalytic core contains 2 identical rings of 7 α-subunits and, between those rings, 2 identical rings of 7 β-subunits. The α-rings mediate the interaction with the 19S regulatory particles and specific α-subunits can also interact with some hydrolases and ubiquitin ligases [21]. Within the β-rings, three β-subunits harbor proteolytic activity: β1 (caspase-like activity), β2 (trypsin-like activity) and β5 (chymotrypsin-like activity), providing a full spectrum of cleavage of peptides after acidic, basic and hydrophobic amino acid residues, respectively. The shorter peptides generated after proteasomal degradation can either be processed for antigen presentation on major histocompatibility complex (MHC) class 1 molecules, or being fully hydrolyzed into amino acids by aminopeptidases and then recycled for protein synthesis [22–24]. Given the high protein turnover and the critical role of the UPS in the development, cell growth and survival of cancer cells [19, 25], proteasome inhibition constitutes an attractive target for chemotherapeutic intervention [4, 26, 27].

In solid tumors, proteasome inhibition will mainly impact the constitutive proteasome. An alternative variant of the constitutive proteasome, i.e., the immunoproteasome, is dominantly expressed in hematopoietic cells wherein constitutive β1, β2 and β5 catalytic subunits are replaced by their β1i, β2i and β5i immunoproteasome counterparts [28]. Whereas solid tumor cells may express low levels of immunoproteasomes, constitutive proteasome expression is most abundant (> 80–90%) and represents the main target for PIs in solid tumors [29, 30].

## Mechanisms of action of proteasome inhibition

Proteasome inhibition by PIs triggers multiple events which contribute to cell death. These events are described in more detail below.

### Endoplasmic reticulum stress and the unfolded protein response

The main mechanism of cell death induction by PIs involves the accumulation of toxic (poly)ubiquitinated proteins and aggregates of misfolded proteins that induce endoplasmic reticulum (ER)-stress. ER-stress initiates the activation of the unfolded protein response (UPR) [14, 31]. There are three ER stress sensors that initiate UPR: PKR-like ER kinase (PERK), inositol requiring kinase 1 (IRE1α), and activating transcription factor (ATF6) [32, 33]. The UPR is coordinated by the activation of these stress sensors, which results in blocking of protein translation, restriction of more unfolded proteins accumulation, activation of genes encoding ER-resident chaperones, and restoration of the folding capacity facilitated by ER-associated degradation (ERAD) [14]. When, upon strong or prolonged ER-stress exposure, the UPR cannot compensate the ER-stress, upregulation of pro-apoptotic proteins facilitates apoptosis induction [14, 31].

The accumulation of unfolded proteins also coincides with the induction of reactive oxygen species (ROS) [34] which activate the caspase cascade and thus contributes to PI-induced apoptosis [35, 36].

### Inhibition of the pro-survival NFκB pathway

NFκB is an inflammation-associated transcription factor that plays a role in the inhibition of apoptosis and in particular in activation of pro-survival pathways. For the activation of NFκB the proteasome is instrumental [19]. PI treatment inhibits the proteasomal degradation of the natural inhibitor of NFκB, IκBα, preventing the nuclear translocation and activation of NFκB. Although originally proposed as a main mechanism of action of PIs [37], it was not a dominant contributor of PI-induced cytotoxicity in multiple myeloma cells [38]. However, as most chemotherapeutics trigger NFκB activation [19, 39], and the fact that specific cancer types are highly dependent on the NFκB pathway for their survival [40–42], this mechanistic feature of PIs can still be very relevant.

### Induction of pro-apoptotic proteins

Since many pro-apoptotic proteins are commonly tagged for degradation or inactivation by the UPS, these proteins can be stabilized upon PI treatment. In many cancer types, P53, a tumor suppressor protein, is inactivated. However, PIs proved to stabilize and reactivate P53, increasing PIs pro-apoptotic effects [39, 43].

Other pro-apoptotic proteins are from the Bcl-2 family. The Bcl-2 family proteins contain anti-apoptotic proteins, e.g., BCL-XI, BCL-2, MCL-1, and pro-apoptotic proteins, e.g., Bax, Bad, Bak, Bim. In cancer cells, pro-apoptotic protein Bim is often degraded by the proteasome, which results in a restriction of Bim’s pro-apoptotic effects. PI treatment stabilizes Bim, and therefore, shifts the balance of the pro- and anti-apoptotic proteins of the Bcl-2 family [44–47].

### Autophagy

Induction of autophagy as alternative pathway for degrading and recycling intracellular proteins may function as pro-survival route upon PI-induced ER-stress [48, 49]. Cytosolic aggregates of ubiquitinated proteins (aggresomes) are transported by microtubules to lysosomes and degraded by autophagy [50, 51]. Type II histone deacetylase (HDAC), plays a crucial role in the microtubule-associated transport of aggresomes as indicated by the fact that a pan HDAC inhibitor like vorinostat, abolished protective autophagy after PI exposure [48, 52–54].

## 26S Proteasome inhibitors

In 2003, BTZ was the first PI approved by the FDA in the US, the EMA authorized BTZ in March 2012. BTZ is a dipeptide boronic acid derivative and a reversible inhibitor of the proteasome that preferentially binds to the β5-subunit. Besides binding to the β5-subunit, BTZ also binds, with a lower affinity, to the β1-subunit (Table 1) [19, 25]. BTZ demonstrated promising results for the treatment of relapsed and refractory MM and was approved for first line MM treatment in 2008. For BTZ combined with dexamethasone (DEX), as first line therapy, response rates of approximately 80% were observed [55–57]. Nonetheless, limiting factors in BTZ therapy included its oral availability, off-target activity and acquired resistance. The most prominent clinical adverse event included peripheral neuropathy, caused by off-target inhibition by BTZ of a neuronal survival protein, HtrA2/Omi [58, 59]. These neurotoxic side effects could be diminished by alternative scheduling and route of administration. BTZ resistance development was recognized as a relevant issue, as both a sub-population of patients had no response to BTZ and a large proportion of patients relapsed on BTZ treatment. To overcome these limiting factors, second-generation PIs were developed to improve the efficacy, reducing the toxicity, enhancing the oral availability and overcoming BTZ resistance by targeting multiple β catalytic subunits and/or do this, other than BTZ, in an irreversible manner (Table 1) [60, 61].

**Table 1:**
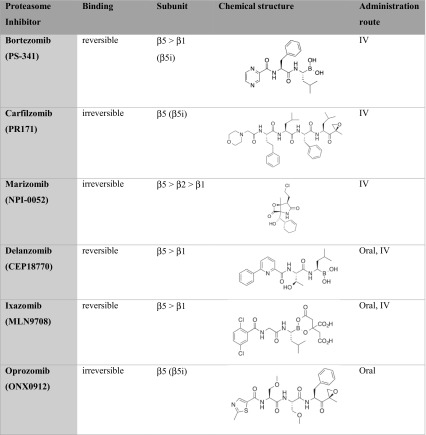
Characteristics of proteasome inhibitors including mode of binding, preferred subunit targeting, chemical structure and administration route

Carfilzomib (CFZ) is the second PI approved by the FDA in 2012 as a 3rd line treatment in MM and by the EMA in 2015 [62]. CFZ is structurally, chemically and mechanistically different from BTZ. Like BTZ, CFZ preferentially binds to the β5-subunit of the proteasome, but does so in an irreversible manner, with greater selectivity and lesser off-target activity, contributing to an improved clinical safety profile as compared to BTZ [63]. Moreover, the covalent binding of CFZ ensures prolonged proteasome inhibition [64]. Lastly, CFZ showed cytotoxic activity against BTZ-resistant cells [65, 66].

Marizomib is a naturally occurring PI derived from the marine actinobacterium *Salinospora tropica* which irreversibly inhibits all three, β1, β2 and β5, subunits [9, 10, 58, 67, 68]. Marizomib has a β-lactone backbone which distinguishes this PI from other clinically active peptide-based PIs [67]. Partly because of the irreversible binding to the various proteasome subunits, marizomib is well retained within cells [69]. However, marizomib has an exceptionally short plasma half-life time of less than 5 min, and a wide tissue distribution. The latter includes penetration of the blood brain barrier which determines it dose limiting toxicity.

Delanzomib is a reversible boronate-based PI, which, other than BTZ, is also orally available [64, 68, 70–72]. The drug preferentially binds to the β5-proteasome subunit and with a lower affinity to the β1-subunit. Delanzomib is active against (BTZ-resistant) MM cells and is less cytotoxic to normal human cells (epithelial cells, bone marrow progenitor or derived stromal cells) [73].

Ixazomib is an orally bioavailable boronic ester prodrug, which reversibly binds to the β5-and β1-subunits [64, 68, 70, 71, 74]. The drug was the first oral PI to enter clinical investigation and is approved for the treatment of MM since September 2016. Ixazomib is clinically active in heavily pre-treated and refractory/relapsed MM, in vitro Ixazomib had the ability to overcome BTZ resistance in MM cells [75].

Finally, oprozomib represents an orally bioavailable PI which irreversibly binds to the β5-subunit. In vitro, the potency of oprozomib is similar to that of CFZ, on top of which oprozomib exhibits activity against BTZ-resistant cells [76–78].

## Proteasome inhibitors in solid malignancies

Building on the success of PIs hematological malignancies, the potential application of PIs in other cancer types has been explored. Hereafter, positioning of PIs for the treatment of most common solid tumor types is discussed.

### Lung cancer

Because of the unfavorable prognosis of lung cancer, the search for new therapies is still indicated and ongoing. Also PIs are being tested for their efficacy in small cell lung cancer (SCLC) and non-small cell lung cancer (NSCLC). BTZ showed encouraging in vitro activity against a panel of human NSCLC cell lines, even though NSCLC cells with aberrant apoptosis (increased Bcl-2) or high basal proteasome activity were intrinsically less sensitive to BTZ [29, 79]. NSCLC cells were also in vitro sensitive to second-generation PIs including CFZ and oprozomib [29]. However, in early clinical trials for NSCLC, BTZ as single agent showed only modest activity [80]. Since studies with NSCLC xenograft models indicated that BTZ pharmacokinetics and tumor penetration were limiting factors determining its efficacy [81], strategies to enhance BTZ efficacy focused on combination therapies, improving tumor penetration and second-generation PIs.

Combination therapies of BTZ with paclitaxel/carboplatin/radiation, irinotecan, radiation and the HDAC inhibitor vorinostat showed promising results in NSCLC therapy [42, 82–85]. However, cisplatin with, or without gemcitabine, did not improve the efficacy of BTZ [86, 87]. Moreover, the addition of BTZ to the current NSCLC chemotherapeutic regimen of gemcitabine and cisplatin did not improve the results of gemcitabine and cisplatin alone [86, 88] even though in vitro studies with NSCLC cells demonstrated a schedule-dependent effect of BTZ increasing the expression of deoxycytidine kinase, the activating enzyme for gemcitabine, and concomitantly levels of the active metabolite of gemcitabine [89]. BTZ was also tested in combination with TRAIL (tumor necrosis factor-related apoptosis-inducing ligand) revealing potent activity against monolayer cultures of NSCLC cells, but had marginal effects in a three-dimensional spheroid NSCLC model [90].

After BTZ treatment, cells accumulate in the radiosensitive G2/M phase of the cell cycle. Moreover, proteasome inhibition disrupts radioresistance mechanisms such as NFkB activation, loss of p53 and DNA DSBs repair [82, 91]. These effects provide a rationale for synergism of BTZ with radiotherapy in lung cancer [82, 85, 91].

Like in NSCLC, BCL-2 overexpression in SCLC is linked to apoptosis-related chemotherapy resistance. In H526 SCLC cell lines, BTZ treatment reduced BCL-2 levels to enhance apoptosis induction and cytotoxicity [92].

The chemotherapeutic effect of the second-generation PIs CFZ has been tested in mouse xenograft model of SHP77 SCLC cells, revealing a significant survival advantage along with a marked increase in cleaved caspase-3 after CFZ treatment [88].

To overcome the poor penetration of BTZ into lung tumors, alternative PI delivery systems were investigated, nanoparticles or micelle formulations [93, 94]. A study by Lin et al. [94] showed that a micelle polymer formulation enhanced the stability of CFZ, allowing a controlled release of CFZ exerting a more potent cytotoxic effect against H460 lung cancer cells compared to free CFZ [94]. Similarly, delivery of BTZ via hollow meoporous silica nanospheres (HMSNs) impacted viability of lung cancer cells to a greater extent than free BTZ [95]. Since about half of NSCLC cells harbor p53 gene mutations, which are associated with poor prognosis [96, 97], the effect of BTZ versus HMSN-BTZ was investigated for wild-type p53 cells compared to mutant p53 cells [95]. Whereas BTZ displayed greater activity against wild-type p53 cells than mutant p53 cells, both cell types gained sensitivity upon HMSN-BTZ treatment. A further gain of HMSN-BTZ sensitivity was observed for mutant p53 cells transfected with wild-type p53 [95]. Given the notion that HMSN-BTZ had a faster release in cancer cells compared to healthy cells, this would favor efficacy and reduce potential side effects.

Together, these results indicate that PIs display activity in lung cancer, however, as a single agent in clinical trials their efficacy is limited. Combination therapies with paclitaxel, carboplatin, irinotecan, vorinostat, gemcitabine, TRAIL and with radiation seem more encouraging. Additional research is necessary to optimize these combined treatments with respect to dose-schedules and improve the clinical effects of PIs by improving its penetration with nano-particles. While most studies focus on NSCLC, SCLC is also eligible for further (clinical) investigation, as well as prognostic factors such as BCL-2 and p53.

### Pancreatic cancer

Pancreatic cancer has a dismal prognosis, with a 5-year survival of less than 5%. Therefore, for this disease, there is an urgent need to identify new chemotherapy regimens, and PI’s are candidates currently being tested. BTZ can induce apoptosis in pancreatic cancer cells via ER-stress [98]. Consistent with data for lung cancer, the combination of PIs with radiation therapy showed synergistic cytotoxic effects in pancreatic cancer as a result of increased ER-stress indicated by enhanced protein levels of IRE1α and JNK [99].

Ninety percent of patients with pancreatic cancer have activating KRAS mutations, which attribute to the poor prognosis. In pancreatic cancer cells, KRAS mutations induce increased levels of the TNF-receptor family member TRAF6, which has a role in maintaining cell survival [99]. Notably, upon PI (BTZ or MG132) treatment TRAF6 undergoes autophagy-dependent rather than proteasome-dependent degradation [99, 100]. The treatment of pancreatic cancer cells with a combination of PIs and radiation resulted in marked TRAF6 downregulation, enhanced autophagy, and increased cytotoxicity [99]. The mechanistic role of TRAF6 in PI-induced autophagy in pancreatic cancer cell death was further supported by the fact that autophagy inhibitors significantly reduced the cytotoxicity [99]. In contrast, a study by Min et al. showed that BTZ induces cell-protective autophagy in primary cultured pancreatic cancer cells and cell lines through activation of AMP-activated protein kinase (AMPK) [101]. AMPK-inhibitors and autophagy inhibitors suppressed autophagy and stimulated BTZ-induced apoptosis of pancreatic cancer cells [101]. The apparent discrepancy in effects of PIs on pancreatic cancer cells could have resulted from different autophagy levels induced by BTZ, in which low and moderate levels of autophagy are protective, while high levels of autophagy promote cell death [102, 103].

A study by Nauman et al. investigated the cytotoxic effects of the PI MG-132 as single agents and in combination with various conventional chemotherapeutics in pancreatic cancer cell lines [104]. Whereas a combination of MG132 and doxorubicin was antagonistic, MG-132 and camptothecin proved synergistic in inducing apoptosis. The latter was confirmed by reduced levels of Mcl-1 protein, an anti-apoptotic protein, and enhanced levels of the pro-apoptotic protein Noxa were found with this combined treatment [104]. The balance of Noxa and Mcl-1 appeared a good indicator for PI-induced apoptosis and could predict the effectivity of PIs in pancreatic cancer cells [104]. Another study showed that BTZ-induced apoptosis in pancreatic cancer cells is associated with the increased production of ceramide lipids [105]. In this regard, Fumonsin B1-induced inhibition of ceramide de novo synthesis decreased BTZ-induced apoptosis and combined treatment of C6-ceramide and BTZ significantly increased cell death of pancreatic cancer cells [105].

While the in vitro results revealed promising activity of PIs in pancreatic cancer cells, one initial clinical study with the PI marizomib in combination with vorinostat, a HDAC inhibitor, showed no clinical responses in patients with pancreatic cancer [106]. However, it might be recommended to combine a PI with one of the standard treatments in pancreatic cancer.

PI-conjugated nanoparticles may be an approach to improve delivery in pancreatic cancer cells and enhance the efficacy in a clinical setting. To this end, BTZ conjugated to pegylated gold nanoparticles showed an enhanced cytotoxicity to pancreatic cancer cell lines as compared to free BTZ [107]. Moreover, conjugated BTZ was less toxic to normal pancreatic cells [107].

Collectively, PIs can induce apoptosis in pancreatic cancer by ER-stress, which facilitates synergistic effects when combined with radiation therapy or drugs like camptothecin. In addition, Mcl-1 and Noxa expression may serve as potential markers for PI activity in pancreatic cancer cells, however, their predictive value needs further clinical confirmation, just as delineating the role of autophagy in pancreatic cancer cells and novel therapeutic approaches with PI-conjugated nanoparticles.

### Breast cancer

Triple-negative breast cancer (TNBC) is a very aggressive form and no specific factors are defined attributing to its poor prognosis. PI’s have been evaluated for their feasibility of breast cancer treatment. Two studies by Tseng et al. and Chen et al. showed anti-tumor activity of PIs in TNBC cells [108, 109]. Marked BTZ- induced apoptosis and anti-tumor activity was noted in TNBC in vitro and in vivo, but not in HER2-overexpressing and estrogen receptor-positive breast cancer cell lines [108]. Notably, BTZ inhibited cancerous inhibitor of protein phosphatase 2A (CIP2A) in the BTZ-sensitive cells [108]. Additionally, several combination therapies were tested to sensitize breast cancer cells to PIs and enhancing their efficacy as described below [109–116]. Lapatinib, a dual EGFR/HER2 tyrosine kinase inhibitor, induced NFkB activation, making the cells more vulnerable for NFkB inhibition, and also induced pro-apoptotic Bax expression in TNBC which resulted in a synergistic anti-tumor activity in vitro and in vivo with BTZ [109]. While BTZ alone did not induce anti-tumor activity in estrogen receptor-positive breast cancer cell lines, the combination treatment of BTZ with anti-estrogens had synergistic effects [116]. Moreover, anti-estrogen resistant breast cancer cells responded to this combined treatment with a decrease in tumor growth [116].

Several combination therapies showed that PIs were able to induce ER-stress in breast cancer cells [110, 112, 113]. Citreoviridin, an ATP synthase inhibitor, triggered PERK-mediated eIF2α phosphorylation, indicating that citreoviridin combined with PIs could increase ER-stress to enhance anti-tumor activity [110]. BTZ was also able to induce apoptosis and autophagy in metastatic breast cancer cell lines [112]. Clarithromycin blocks autophagy flux, and in combination with BTZ significantly enhanced the activation of pro-apoptotic transcription factor CHOP and cytotoxicity in this metastatic breast cancer cell line [112]. In support of this mechanism, a decrease in BTZ-induced cell death was found after knockdown of CHOP [112]. Another combination therapy that induces ER-stress and enhances the cytotoxicity to breast cancer cells is BTZ with vinorelbine (VNR), a suppressor of aggresome formation induced by BTZ [113].

Doxorubicin is an effective chemotherapeutic drug in breast cancer treatment. However, its efficacy is limited by resistance and side effects. Doxorubicin induces activation of NFkB, which could contribute to its resistance. CFZ inhibited NFkB activation and showed cytotoxicity in breast cancer cells [114]. Moreover, CFZ increased doxorubicin-induced apoptosis and cytotoxic effects [114]. Another second-generation PI, ixazomib, also showed cytotoxic effects and enhanced JNK and p38 phosphorylation induced by doxorubicin, sensitizing breast cancer cells to doxorubicin [115].

Together, BTZ has single agent activity against TNBC in vitro and in vivo, and displays synergistic effects when combined with anti-estrogens in estrogen receptor-positive breast cancer. PI-induction of ER-stress in breast cancer cells provides a rationale for evaluating BTZ or ixazomib in combination with drugs currently used breast cancer treatment.

### Head and neck squamous cell carcinoma

Head and neck squamous cell carcinoma (HNSCC) has often an aggressive course with emerging resistance to conventional chemotherapy. Therefore, new agents are under investigation to improve the outcome. Pre-clinical studies showed that BTZ as a monotherapy induced apoptosis in HNSCC cells in vitro and in vivo [117, 118]. Mechanistically, inhibition of CIP2A was largely responsible for apoptosis induction by BTZ in HNSCC [117]. Consistently, HNSCC cells were protected against BTZ-induced apoptosis with overexpression of CIP2A [117]. BTZ also promoted apoptosis and cell cycle arrest in human papillomavirus (HPV) positive HNSCC [118]. Other than non-HPV-positive HNSCC, HPV-positive HNSCC contains wild-type p53, that is rapidly degraded by the proteasome, and could thus be a target for therapy [118]. After BTZ treatment of HPV-positive HNSCC, functional p53 was enhanced, resulting in cell cycle arrest and apoptosis [118]. Second-generation PIs may elicit activity against HNSCC as illustrated by suppression of HNSCC xenograft tumor growth by oprozomib [119].

Despite encouraging pre-clinical results, a phase II clinical trial with single BTZ in HNSCC showed a poor response rate of only 3% [120]. Next, combination therapies were tested to improve the efficacy of PIs in HNSCC [119, 121, 122]. One approach could be to trigger apoptosis with TRAIL [90]. Indeed, in HNSCC, the combination of MG-132 and TRAIL appeared synergistic in inducing apoptosis and cell death as a result of truncated Bid and Bik accumulation [121]. Additionally, CFZ and oprozomib induced apoptosis in HNSCC cells through enhanced Bik [119]. However, these PIs also increased Mcl-1 in HNSCC, thereby decreasing cytotoxic effects [119]. In HNSCC, activation of UPR was also observed which induced protective autophagy [119, 122]. Therefore, suppression of Mcl-1 or autophagy could be strategies to enhance cytotoxic effects. To this end, combining a HDAC inhibitor to BTZ resulted in a decrease of autophagy and a significant increase of apoptosis in HNSCC cells [123].

Summarizing, although BTZ confers single agent activity against HNSCC cells in vitro and in vivo by inhibition of CIP2A, clinical trials with BTZ in head and neck cancer showed poor results, conceivably by induction of protective autophagy and overexpression of CIP2A. Complementary research with respect to factors such as CIP2A, p53, Bik, and Mcl-1 may reveal therapeutic options for combination therapies with, e.g., TRAIL or HDAC inhibitors in head and neck cancer.

### Thyroid cancer

Anaplastic thyroid carcinoma (ATC) has a poor overall survival. This might be due to the absence of thyroid-specific gene expression and refractoriness to the current therapeutic approaches. Therefore, experimental therapeutics for thyroid cancer also included PIs [124–127]. BTZ displayed anti-tumor activity in ATC cells in vitro and in vivo by impairment of glucose metabolism, induction of apoptosis, G2/M cell cycle arrest, and growth inhibition [125]. MG132 induced apoptosis as well as accumulation of p53 in both wt p53 and mut p53 thyroid cancer cells [127]. However, only in ATC cell lines with wt p53, PIs induced upregulation of the pro-apoptotic targets, not in the mut p53 cells [127]. In contrast, pro-apoptotic targets regulated by the tumor suppressor, transcription factor and proteasome substrate forkhead BOX O3 (FOXO3a) were enhanced upon PI exposure in both wt p53 and mut p53, thus triggering increased apoptosis in ATC cells [127].

Studies by Zhang et al. showed that PI treatment of ATC cells decreased the expression of Beclin 1, an autophagy essential protein [128]. Whereas knockdown Beclin 1 did not impact PI cytotoxicity, overexpression of Beclin 1 increased the anti-tumor effects of PIs in ATC cells [128].

The second-generation PI CFZ has also been tested in ATC [124, 126]. CFZ, in direct comparison with BTZ and ixazomib, and was most effective against ATC cells in vitro and in vivo by inducing G2/M cell cycle arrest, as well as apoptosis [124]. Notably, CFZ significantly increased the overall survival in metastatic mice, without significant ADRs [124].

As for the other tumor types, combination therapies with PIs were also tested in ATC [126, 128]. A synergistic activity was found with the combination of CFZ with CUDC-101, a histone deacetylase and multi-kinase inhibitor, due to an increased caspase 3/7 activity and G2/M cell cycle arrest [126].

Together, BTZ showed in vitro and in vivo anti-tumor activity in ATC cells, which was very much p53 status and autophagy-dependent. The role of these two factors deserves further investigation for PI targeting of ATC, for which CFZ seems an attractive candidate.

### Miscellaneous

Beyond the common solid malignancies described above, anti-tumor activity of PIs has also been explored in other solid tumor types, including hepatocellular carcinoma (HCC), oral squamous cell carcinoma, prostate cancer, colorectal cancer, ovarian cancer, biliary tract cancer and melanoma [101, 129–144]. The current status of PIs application in hepatocellular carcinoma was recently reviewed by Chen et al. and showed no clinical effectivity with BTZ, as a single agent, but noteworthy progress is made in identifying and developing UPS-targeting molecules feasible for HCC treatment, yet to enter clinical trials [129]. In most other solid malignancies PIs exerted mechanisms of action similar as described above, i.e., inducing apoptosis, decreasing tumor growth and synergistic activity in some combination therapies [133–136]. These findings suggest that PI treatment of various solid malignancies is feasible, but requires more research to understand, for each tumor types, its full mechanism of action, and identify most promising combination therapies.

Table 2 summarizes currently completed, but not yet published, clinical trials of PIs in solid malignancies.

**Table 2:** Completed trials with PIs in solid tumors (clinicaltrials.gov)

Compound	Combination	Condition	Phase	ClinicalTrials.gov identifier
PS-341	Doxarubicin	Advanced solid tumors	Phase I	NCT00023855
PS-341	Chemotherapy	Advanced solid tumors	Phase I	NCT00028587
PS-341	Topotecan	Advanced malignancies	Phase I	NCT00068484
NPI-0052	–	Solid malignancies or refractory lymphoma	Phase I	NCT00396864
PS-341	–	Advanced or metastatic solid tumors	Phase I	NCT02220049
PS-341	Sorafenib	Advanced cancers	Phase I	NCT00303797
PS-341	–	Children with refractory solid tumors	Phase I	NCT00021216
NPI-0052	–	Advanced malignancies	Phase I	NCT00629473
PS-341	Paclitaxel	Locally advanced or metastatic solid tumor	Phase I	NCT00030368
PS-341	Doxorubicin	Advanced adenoid cystic carcinoma of the head and neck	Phase II	NCT00077428
PS-341	–	Advanced malignancies and kidney dysfunction	Phase I	NCT00054483

## Resistance and predictive factors in solid malignancies

Mechanisms of intrinsic and acquired resistance to PIs in hematological malignancies have been extensively reviewed [8, 10, 145–147]. Here we primarily focus on mechanisms that contribute to PI-resistance in solid malignancies.

### Proteasome activity

Several studies showed that BTZ-resistant cells had higher (basal) proteasome activity and increase in subunit gene expression, compared to BTZ-sensitive cells [29, 79, 148]. This enabled cells a faster recovery of proteasome activity after BTZ treatment thus attenuating BTZ response [148]. Other than with the reversible PI inhibitor BTZ, proteasome inhibition is more prolonged by the irreversible PI CFZ, which contributes to its activity against BTZ-resistant cells [148]. A recent study by Weyburne et al. showed that after β5-subunit inhibition, activation of Nrf1 was mainly responsible for initiating proteasome activity recovery. Interestingly, this process could be blocked by co-inhibition of the β2-subunit [149].

Besides the constitutive proteasome, most commonly expressed in solid tumor cells, cells of the immune system harbor a relatively high expression of immunoproteasomes. Herein the immunoproteasome subunits LMP2 (β1i), MECL1(β2i), and LMP7 (β5i) have replaced the constitutive proteasome subunits β1, β2, and β5. Upon oxidative stress or inflammatory stimuli such as interferon γ (IFNγ) or TNFα, these immunoproteasome subunits can be expressed in other cells as well. In leukemia, there are indications that a higher ratio of immunoproteasomes over constitutive proteasomes is associated with a better response to PIs [150]. A study by Busse et al. compared neoplastic B-cells to several solid tumor cells for their BTZ sensitivity. They found that the solid tumor cells were intrinsically more resistant to BTZ, and had a lower expression of the β1i, β2i, β5i, and β2 subunits, compared to the neoplastic B cells [30]. Moreover, after IFNγ pretreatment, BTZ sensitivity increased in 50% of cell lines [30]. This suggests that a lower expression of the immunoproteasome contributes to PI-resistance in solid malignancies versus hematological malignancies. Remarkably, in two studies acquired resistance to BTZ in solid tumor cell lines coincided with an upregulation of both constitutive and immunoproteasome subunits [29, 148]. The latter most likely represents a compensatory mechanism for malfunctioning β5 subunits due to *PSMB5* mutations (see below).

### Proteasome β5-subunit mutations

*PSMB5* mutations introducing amino acid substitutions in a highly conserved substrate/inhibitor binding domain β5 subunit result in impaired BTZ binding and has been identified as a mechanism of PI-resistance in heamatological malignancies [8, 145]. Also in BTZ-resistant solid tumor cell lines, point mutations in *PSMB5* were identified [29, 148]. A study by De Wilt et al. in lung cancer cells with acquired BTZ resistance revealed Ala49Thr, Met45Val, and Cys52Phe substitutions in the β5 subunit BTZ-binding pocket, while Suzuki et al., in BTZ-resistant colon cancer cells observed Cys63Phe and Arg24Cys mutations in the β5 subunit [29, 148]. BTZ-resistant solid tumor cells displayed cross-resistance to all PIs that target the β5-subunit, but retained sensitivity for PIs targeting other, e.g., α-subunits [29].

### Apoptosis-mediated resistance; Noxa/Mcl-1

In some BTZ-resistant cells an altered Mcl-1/Noxa balance was noted as an attributing factor [29, 39, 104, 151]. In BTZ-resistant melanoma cells, expression of anti-apoptotic protein Mcl-1 was markedly increased after BTZ treatment, whereas expression of pro-apoptotic protein Noxa was unaffected [151]. The BTZ-resistant cells could be sensitized for BTZ-induced apoptosis when induction of Mcl-1 was prevented by Mcl-1 siRNA [151]. Also, in pancreatic cancer cells the Noxa/Mcl-1 balance constitutes a (predictive) factor determining BTZ sensitivity [104, 152]. Finally, NSCLC cell lines with overexpression of the anti-apoptotic Bcl-2 protein, also proved to be more resistant to PI-induced apoptosis [79].

### Autophagy

It is still unclear whether autophagy limits or promotes cell survival. Low or moderate levels of autophagy appear cell-protective, while high levels of autophagy facilitate promotion of cell death [102, 103]. Notwithstanding these facts, multiple studies showed that inhibition of autophagy increased PI-induced apoptosis [39, 101, 122, 134, 153]. Upon PI treatment, protective autophagy was activated in several solid malignancies [101, 122]. A study by Min et al. revealed that the mechanism underlying protective autophagy in BTZ-treated colon and pancreatic cancer cells involved activation of AMP-activated protein kinase (AMPK). Inhibition of autophagy with 3-methyladenine (3-MA) enhanced BTZ-induced cytotoxicity and apoptosis in these cells [101].

Another combination therapy that reduced PI-induced protective autophagy was noted for a histone deacetylase 6 (HDAC6) inhibitor. This combination increased BTZ-induced apoptosis and reversed PI-resistance [39, 122, 153].

### KRAS

Studies by Chattopadhyay et al. examining the sensitivity of the novel PI Ixazomib in a panel of colon cancer and NSCLC xenografts, revealed that tumors harboring activating KRAS mutations were less sensitive to Ixazomib than tumors with wt KRAS [139]. Moreover, introducing activating KRAS mutations into wt KRAS cells markedly reduced Ixazomib sensitivity in these xenograft models [139]. The underlying mechanism of how activating KRAS mutations impact PI sensitivity appears associated with reprogramming key metabolic pathways and the ability of PIs to inhibit these pathways in solid tumors. Given the fact that alterations in metabolic pathways in KRAS-wt and KRAS-mt may differ in tumor cells with different genetic backgrounds, this may also attribute to differential PI sensitivities in solid tumors [139].

### Pgp

**Fig. 2:**
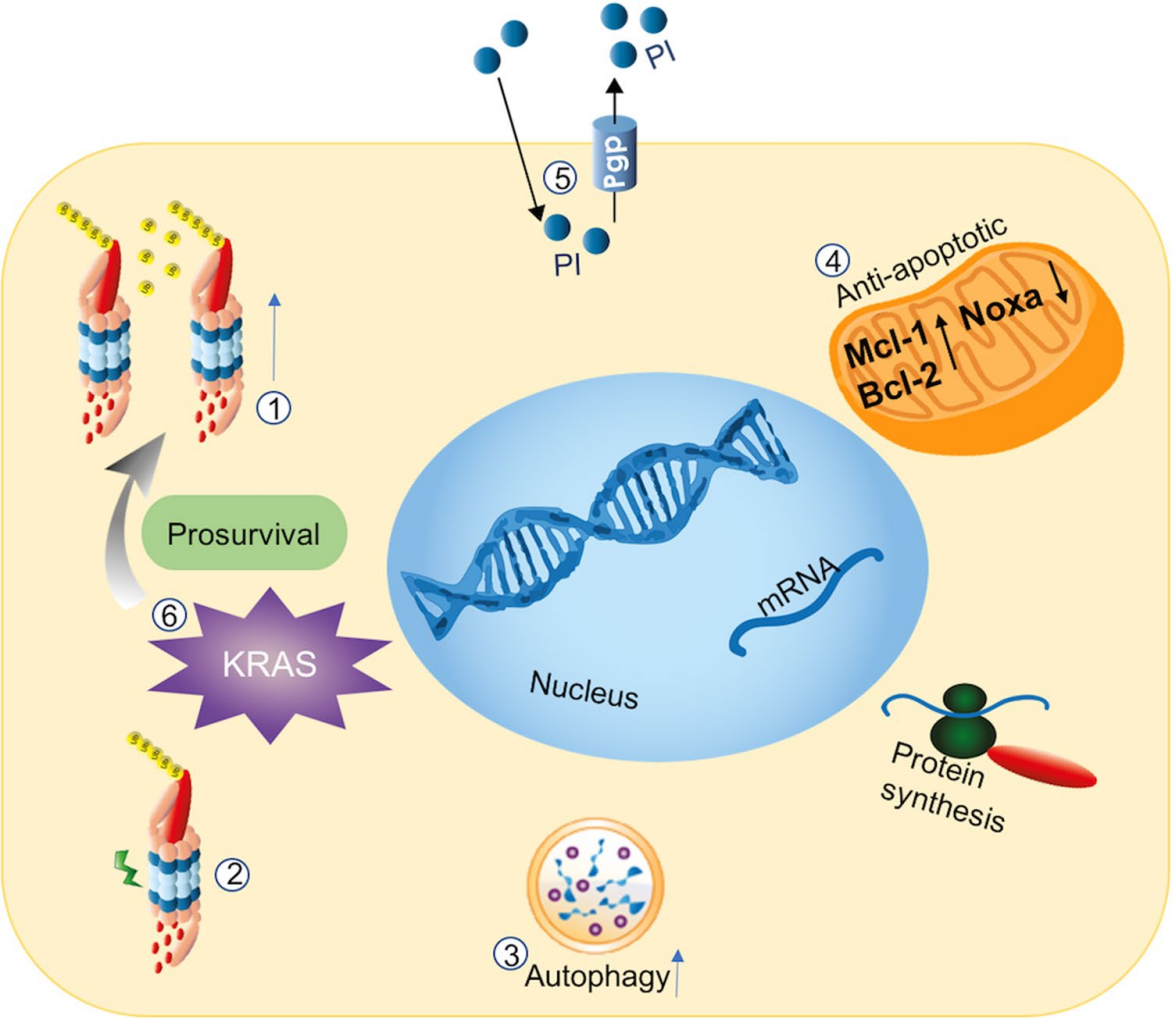
An overview of PI-resistance in solid tumors: *1* upregulation of proteasome activity and increased subunit gene expression, *2* proteasome β5-subunit mutations, *3* protective autophagy, *4* apoptosis-mediated resistance due to an altered Mcl-1/Noxa balance, *5* elevated levels of Pgp resulting in an enhanced CFZ efflux, and *6* KRAS mutations associated with reprogramming metabolic pathways

Elevated levels of the multidrug efflux transporter P-glycoprotein (Pgp) were observed in human colon, lung, and head and neck squamous cell carcinoma cell lines with acquired resistance to CFZ [153, 154]. The notion that inhibitors of Pgp were able to reverse CFZ resistance indicates that enhanced Pgp-mediated drug efflux can confer CFZ resistance [154]. Indeed, a study by Verbrugge et al. demonstrated that CFZ and oprozomib were bona fide substrates for Pgp and thus Pgp-overexpression facilitated resistance to these PIs [155]. BTZ is a poor substrate for Pgp and the efflux transporter does not play a dominant role in BTZ resistance [155, 156]. Studies by Verbrugge et al. also documented that BTZ and another second-generation PIs were non-substrates for other family members of ATP-driven drug efflux transporters, and consistently, did not play a role in PI resistance [155]. A summarizing graphical presentation of PI-resistance modalities in solid tumors as shown in Fig. 2.

## Discussion

The level of success of PI treatment for hematological malignancies has thus far not been reached for solid tumors. Despite encouraging activity and anti-tumor effects of PIs in pre-clinical models of solid tumors, their clinical activity is still limited and requires additional mechanistic insights to improve on this. Recognizing that PIs have overlapping mechanisms of action in hematological malignancies versus solid tumors, research has also pinpointed critical differences, which collectively attribute to their differential response to PIs. These include differences in proteasome subunit composition, drug penetration, the impact of oncogene activation, autophagy and apoptosis induction, and resistance mechanisms.

PI targeting in solid tumors mainly involves constitutive proteasomes, while in hematological malignancies immunoproteasomes are much more abundant and constitute an additional target on top of constitutive proteasomes. Clinically directed PI research in solid tumors would greatly benefit from quantitative and qualitative assessments of constitutive and immunoproteasome subunit composition in clinical samples of various types of solid tumors. Analytical methods are available which can provide detailed information on proteasome subunit composition from limited (biopsy) sample sizes and thus help in designing rationalized PI treatment strategies [157, 158]. Although the abundance of immunoproteasomes in solid tumors is estimated to be low, emerging functions of immunoproteasomes in handling oxidative and toxic stress could hold relevance for PI (combination) therapies in solid tumors [159, 160]. Inhibition this function may account for the fact-specific immunoproteasome inhibitors conferred growth inhibitory effects against lung cancer cell lines [29, 161]. In cases of inflammation-driven cancers, the release of TNFα and IFN-γ may further enhance the expression of immunoproteasomes to levels that would give grounds for PI targeting.

Drug penetration in solid tumors was recognized as a factor limiting PI efficacy. From this perspective, a rationalized choice for irreversible PIs (CFZ, oprozomib) over reversible PIs (BTZ, ixazomib) could be made to achieve a better retention and prolong proteasome inhibition. However, since CFZ and oprozomib are substrates for the drug efflux transporter Pgp, this could potentially interfere with drug penetrations [155]. To further improve on drug penetration, early results demonstrated the feasibility of alternative PI delivery methods, e.g., via nanoparticles, and require follow up studies. Beyond penetration, also hypoxic conditions can impair drug activity, although under severe hypoxia in vitro, carcinoma cells were fully sensitive for BTZ when compared to normoxic conditions [162].

The exact role of autophagy in PI response needs further studies as to its relevance for selected or most solid tumor types. The same holds for the impact of the genetic background and oncogenic expression/activation in various solid tumor types in relation to PI response. The outcome of these studies may provide better rationales for combination studies of PIs with radiotherapy, HDAC inhibitors and TRAIL which have shown to be effective in selected tumor types.

Finally, as to the issue of drug resistance to PIs in solid tumors, overlapping mechanisms with PI-resistance in hematological malignancies were identified [8]. These mechanisms, however, were mostly derived from solid tumor cell line studies and need confirmation in clinical specimen from patients with solid tumor refractory to PI treatment. One recently identified novel mechanism of BTZ resistance in leukemia may also be of interest to explore in solid tumors. This relates to the role of the Myristoylated Alanine-Rich C Kinase Substrate (MARCKS) protein in vesicular/exosome-mediated exocytosis of ubiquitinated proteins from BTZ-resistant cells to quench proteolytic stress [163]. Since MARCKS protein is also abundantly expressed in lung tissue and lung cancer, it may also provide a potential mechanism of resistance to BTZ in lung cancer [164, 165]. A role of MARCKS protein in BTZ resistance was originally indicated from proteomic and differential gene expression profiling studies in BTZ-resistant hematological cells [163, 166]. Given that most characterizations of the effects of PIs on solid tumors relied on assessments of apoptosis induction and cell cycle effects, application of these novel technologies can aid to further pinpoint other critical processes that determine PI sensitivity/resistance in solid tumors. In this respect, also metabolomics may be explored as an entity impacted by PIs. Notably, BTZ resistance in MM cell lines and patient samples has been associated with a reprogrammed glucose metabolism [167]. In fact, BTZ-resistant cells featured a higher activity of the serine synthesis pathway, and interestingly serine starvation provoked increased BTZ-cytotoxicity [167]. Metabolic aberrations might also be important in solid malignancies since KRAS mutations found in resistant colon cancer and NSCLC xenografts were associated with reprogrammed metabolic pathways. Anticipating rewiring of metabolic pathways (e.g., glucose and amino acid metabolism) in PI-resistant cells, interfering with these pathways might constitute novel strategies to enhance PI’s cytotoxicity in combination therapies [22].

Beyond evaluating next generation PI inhibitors, strategies to overcome BTZ resistance mechanisms deserve focus on exploring PIs that target non-catalytic subunits of the proteasome or other targets in the UPS, e.g., deubiquitinases [168, 169].

Collectively, identifications of novel determinants of PI sensitivity/resistance in solid tumors, employing advanced genetic, proteomic and metabolomics techniques, will be helpful to guide future rational strategies single agent PI or PI combination therapies to improve therapy clinical efficacy.

## References

[1] Wijdeven RH, Pang B, Assaraf YG, Neefjes J (2016). Old drugs, novel ways out: drug resistance toward cytotoxic chemotherapeutics. Drug Resist Updat.

[2] Dou QP, Zonder JA (2014). Overview of proteasome inhibitor-based anti-cancer therapies: perspective on bortezomib and second generation proteasome inhibitors versus future generation inhibitors of ubiquitin-proteasome system. Curr Cancer Drug Targets.

[3] Hochstrasser M (1995). Ubiquitin, proteasomes, and the regulation of intracellular protein degradation. Curr Opin Cell Biol.

[4] Dou QP, Li B (1999). Proteasome inhibitors as potential novel anticancer agents. Drug Resist Updat.

[5] Anderson KC (2012). The 39th David A. Karnofsky Lecture: bench-to-bedside translation of targeted therapies in multiple myeloma. J Clin Oncol.

[6] San-Miguel JF, Mateos MV (2011). Can multiple myeloma become a curable disease?. Haematologica.

[7] Cloos J, Roeten MSF, Franke NE (2017). (Immuno) proteasomes as therapeutic target in acute leukemia. Cancer Metastasis Rev.

[8] Niewerth D, Jansen G, Assaraf YG (2015). Molecular basis of resistance to proteasome inhibitors in hematological malignancies. Drug Resist Updat.

[9] Huber EM, Heinemeyer W, Groll M (2015). Bortezomib-resistant mutant proteasomes: Structural and biochemical evaluation with carfilzomib and ONX 0914. Structure.

[10] Niewerth D, Jansen G, Riethoff LF (2014). Antileukemic activity and mechanism of drug resistance to the marine *Salinispora tropica* proteasome inhibitor salinosporamide A (Marizomib). Mol Pharmacol.

[11] Corso A, Mangiacavalli S, Varettoni M (2010). Bortezomib-induced peripheral neuropathy in multiple myeloma: a comparison between previously treated and untreated patients. Leuk Res.

[12] Cavaletti G, Jakubowiak AJ (2010). Peripheral neuropathy during bortezomib treatment of multiple myeloma: a review of recent studies. Leuk Lymphoma.

[13] Huang Z, Wu Y, Zhou X (2014). Efficacy of therapy with bortezomib in solid tumors: a review based on 32 clinical trials. Future Oncol.

[14] Ri M (2016). Endoplasmic-reticulum stress pathway-associated mechanisms of action of proteasome inhibitors in multiple myeloma. Int J Hematol.

[15] Ciechanover A (1994). The ubiquitin-proteasome proteolytic pathway. Cell.

[16] Demarchi F, Brancolini C (2005). Altering protein turnover in tumor cells: New opportunities for anti-cancer therapies. Drug Resist Updat.

[17] Komander D, Clague MJ, Urbé S (2009). Breaking the chains: structure and function of the deubiquitinases. Nat Rev Mol Cell Biol.

[18] Streich FC, Lima CD (2014). Structural and functional insights to ubiquitin-like protein conjugation. Annu Rev Biophys.

[19] Adams J (2004). The proteasome: a suitable antineoplastic target. Nat Rev Cancer.

[20] Glickman MH, Ciechanover A (2002). The ubiquitin-proteasome proteolytic pathway: destruction for the sake of construction. Physiol Rev.

[21] Moiseeva TN, Bottrill A, Melino G, Barlev NA (2013). DNA damage-induced ubiquitylation of proteasome controls its proteolytic activity. Oncotarget.

[22] Hitzerd SM, Verbrugge SE, Ossenkoppele G (2014). Positioning of aminopeptidase inhibitors in next generation cancer therapy. Amino Acids.

[23] Reits E, Griekspoor A, Neijssen J (2003). Peptide diffusion, protection, and degradation in nuclear and cytoplasmic compartments before antigen presentation by MHC class I. Immunity.

[24] Rock K, York I, Saric T, Goldberg A (2002). Protein degradation and the generation of MHC class I-presented peptides. Adv Immunol.

[25] Chen D, Frezza M, Schmitt S (2011). Bortezomib as the first proteasome inhibitor anticancer drug: current status and future perspectives. Curr Cancer Drug Targets.

[26] Almond JB, Cohen GM (2002). The proteasome: a novel target for cancer chemotherapy. Leukemia.

[27] Manasanch EE, Orlowski RZ (2017). Proteasome inhibitors in cancer therapy. Nat Rev Clin Oncol.

[28] Groettrup M, Kirk CJ, Basler M (2010). Proteasomes in immune cells: more than peptide producers?. Nat Rev Immunol.

[29] de Wilt LH, Jansen G, Assaraf YG (2012). Proteasome-based mechanisms of intrinsic and acquired bortezomib resistance in non-small cell lung cancer. Biochem Pharmacol.

[30] Busse A, Kraus M, Na IK (2008). Sensitivity of tumor cells to proteasome inhibitors is associated with expression levels and composition of proteasome subunits. Cancer.

[31] Obeng EA, Carlson LM, Gutman DM (2006). Proteasome inhibitors induce a terminal unfolded protein response in multiple myeloma cells. Blood.

[32] Kaufman RJ (2002). Orchestrating the unfolded protein response in health and disease. J Clin Invest.

[33] Ron D (2002). Translational control in the endoplasmic reticulum stress response. J Clin Invest.

[34] Shimizu Y, Hendershot LM (2009). Oxidative folding: cellular strategies for dealing with the resultant equimolar production of reactive oxygen species. Antioxid Redox Signal.

[35] Fribley A, Zeng Q, Wang C (2004). Proteasome inhibitor PS-341 induces apoptosis through induction of endoplasmic reticulum stress-reactive oxygen species in head and neck squamous cell carcinoma cells. Mol Cell Biol.

[36] Pérez-Galán P, Roue G, Villamor N (2006). The proteasome inhibitor bortezomib induces apoptosis in mantle-cell lymphoma through generation of ROS and Noxa activation independent of p53 status. Blood.

[37] Paramore A, Frantz S (2003). Bortezomib. Nat Rev Drug Discov.

[38] Hideshima T, Ikeda H, Chauhan D (2009). Bortezomib induces canonical nuclear factor-kappaB activation in multiple myeloma cells. Blood.

[39] McConkey DJ, Zhu K (2008). Mechanisms of proteasome inhibitor action and resistance in cancer. Drug Resist.

[40] Chaturvedi MM, Sung B, Yadav VR (2011). NFkB addiction and its role in cancer : “one size does not fit all. ” Oncogene.

[41] Nencioni A, Grünebach F, Patrone F (2007). Proteasome inhibitors: antitumor effects and beyond. Leukemia.

[42] Gatti L, Zuco V, Zaffaroni N, Perego P (2013). Drug combinations with proteasome inhibitors in antitumor therapy. Curr Pharm Des.

[43] Rastogi N, Duggal S, Singh SK (2015). Proteasome inhibition mediates p53 reactivation and anti- cancer activity of 6-Gingerol in cervical cancer cells. Oncotarget.

[44] Li C, Li R, Grandis JR, Johnson DE (2008). Bortezomib induces apoptosis via Bim and Bik up-regulation and synergizes with cisplatin in the killing of head and neck squamous cell carcinoma cells. Mol Cancer Ther.

[45] Tan T, Degenhardt K, Nelson DA (2005). Key roles of BIM-driven apoptosis in epithelial tumors and rational chemotherapy. Cancer Cell.

[46] Wirth M, Stojanovic N, Christian J (2014). MYC and EGR1 synergize to trigger tumor cell death by controlling NOXA and BIM transcription upon treatment with the proteasome inhibitor bortezomib. Nucleic Acids Res.

[47] Fennell DA, Chacko A, Mutti L (2008). BCL-2 family regulation by the 20S proteasome inhibitor bortezomib. Oncogene.

[48] Ding W-X, Ni H-M, Gao W (2007). Linking of autophagy to ubiquitin-proteasome system is important for the regulation of endoplasmic reticulum stress and cell viability. Am J Pathol.

[49] Ding WX, Ni HM, Gao W (2007). Differential effects of endoplasmic reticulum stress-induced autophagy on cell survival. J Biol Chem.

[50] Nawrocki ST, Carew JS, Pino MS (2006). Aggresome disruption: a novel strategy to enhance bortezomib- induced apoptosis in pancreatic cancer cells. Cancer Res.

[51] Simms-waldrip T, Rodriguez-gonzalez A, Lin T (2008). The aggresome pathway as a target for therapy in hematologic malignancies. Mol Genet Metab.

[52] Pandey UB, Nie Z, Batlevi Y (2007). HDAC6 rescues neurodegeneration and provides an essential link between autophagy and the UPS. Nature.

[53] Tallóczy Z, Jiang W, Virgin HW (2002). Regulation of starvation- and virus-induced autophagy by the eIF2alpha kinase signaling pathway. Proc Natl Acad Sci USA.

[54] Carew JS, Medina EC, Esquivel JA (2010). Autophagy inhibition enhances vorinostat-induced apoptosis via ubiquitinated protein accumulation. J Cell Mol Med.

[55] Harousseau JL, Attal M, Avet-Loiseau H (2010). Bortezomib plus dexamethasone is superior to vincristine plus doxorubicin plus dexamethasone as induction treatment prior to autologous stem-cell transplantation in newly diagnosed multiple myeloma: results of the IFM 2005–01 phase III trial. J Clin Oncol.

[56] Jagannath S, Durie BGM, Wolf J (2005). Bortezomib therapy alone and in combination with dexamethasone for previously untreated symptomatic multiple myeloma. Br J Haematol.

[57] Jagannath S, Durie BGM, Wolf JL (2009). Extended follow-up of a phase 2 trial of bortezomib alone and in combination with dexamethasone for the frontline treatment of multiple myeloma. Br J Haematol.

[58] Arastu-Kapur S, Anderl JL, Kraus M (2011). Nonproteasomal targets of the proteasome inhibitors bortezomib and carfilzomib: a link to clinical adverse events. Clin Cancer Res.

[59] Voortman J, Giaccone G (2007) Clinical application of proteasome inhibitor bortezomib : characterization of neurotoxicity. Ubiquitin proteasome Syst. Cent. Nerv. Syst. from Physiol. to Pathol. pp 1037–1054

[60] Allegra A, Alonci A, Gerace D (2014). New orally active proteasome inhibitors in multiple myeloma. Leuk Res.

[61] Moreau P, Richardson PG, Cavo M (2015). Review article Proteasome inhibitors in multiple myeloma: 10 years later. Blood.

[62] Kortuem KM, Stewart AK (2013). Carfilzomib Blood.

[63] Siegel D, Martin T, Nooka A (2013). Integrated safety profile of single-agent carfilzomib: experience from 526 patients enrolled in 4 phase II clinical studies. Haematologica.

[64] Ruschak AM, Slassi M, Kay LE, Schimmer AD (2011). Novel proteasome inhibitors to overcome bortezomib resistance. J Natl Cancer Inst.

[65] Demo SD, Kirk CJ, Aujay MA (2007). Antitumor activity of PR-171, a novel irreversible inhibitor of the proteasome. Cancer Res.

[66] Kuhn DJ, Chen Q, Voorhees PM (2007). Potent activity of carfilzomib, a novel, irreversible inhibitor of the ubiquitin-proteasome pathway, against preclinical models of multiple myeloma. Blood.

[67] Potts C, Albitar BX, Anderson MC (2011). Marizomib, a proteasome inhibitor for all seasons: preclinical profile and a framework for clinical trials. Curr Cancer Drug Targets.

[68] Kisselev AF, Van Der Linden WA, Overkleeft HS (2012). Proteasome inhibitors: An expanding army attacking a unique target. Chem Biol.

[69] Obaidat A, Weiss J, Wahlgren B (2011). Proteasome regulator marizomib (NPI-0052) exhibits prolonged inhibition, attenuated efflux, and greater cytotoxicity than its reversible analogs. J Pharmacol Exp Ther.

[70] Dick LR, Fleming PE (2010). Building on bortezomib: second-generation proteasome inhibitors as anti-cancer therapy. Drug Discov Today.

[71] Buac D, Shen M, Schmitt S (2013). From bortezomib to other inhibitors of the proteasome and beyond. Curr Pharm Des.

[72] Piva R, Ruggeri B, Williams M (2008). CEP-18770: a novel, orally active proteasome inhibitor with a tumor-selective pharmacologic profile competitive with bortezomib. Blood.

[73] Berkers CR, Leestemaker Y, Schuurman KG (2012). Probing the specificity and activity profiles of the proteasome inhibitors bortezomib and delanzomib. Mol Pharm.

[74] Kouroukis CT, Fernandez LA, Crump M (2011). A phase II study of bortezomib and gemcitabine in relapsed mantle cell lymphoma from the National Cancer Institute of Canada Clinical Trials Group (IND 172). Leuk Lymphoma.

[75] Chauhan D, Tian Z, Zhou B (2011). In vitro and in vivo selective antitumor activity of a novel orally bioavailable proteasome inhibitor MLN9708 against multiple myeloma cells. Clin Cancer Res.

[76] Chauhan D, Singh AV, Aujay M (2010). A novel orally active proteasome inhibitor ONX 0912 trigger in vitro and in vivo cytotoxicity in multiple myeloma. Blood.

[77] Nooka A, Gleason C, Casbourne D, Lonial S (2013). Relapsed and refractory lymphoid neoplasms and multiple myeloma with a focus on carfilzomib. Biol Targets Ther.

[78] Thompson JL (2013). Carfilzomib: a second-generation proteasome inhibitor for the treatment of relapsed and refractory multiple myeloma. Ann Pharmacother.

[79] Voortman J, Checińska A, Giaccone G (2007). The proteasomal and apoptotic phenotype determine bortezomib sensitivity of non-small cell lung cancer cells. Mol Cancer.

[80] Fanucchi MP, Fossella FV, Belt R (2006). Randomized phase II study of bortezomib alone and bortezomib in combination with docetaxel in previously treated advanced non-small-cell lung cancer. J Clin Oncol.

[81] Williamson MJ, Silva MD, Terkelsen J (2009). The relationship among tumor architecture, pharmacokinetics, pharmacodynamics, and efficacy of bortezomib in mouse xenograft models. Mol Cancer Ther.

[82] Zhao Y, Foster NR, Meyers JP (2015). A phase I/II study of bortezomib in combination with paclitaxel, carboplatin, and concurrent thoracic radiation therapy for non-small-cell lung cancer. J Thorac Oncol.

[83] Jones DR, Moskaluk CA, Gillenwater HH (2012). Phase I trial of induction histone deacetylase and proteasome inhibition followed by surgery in non-small-cell lung cancer. J Thorac Oncol.

[84] Arnold SM, Chansky K, Leggas M (2017). Phase 1b trial of proteasome inhibitor carfilzomib with irinotecan in lung cancer and other irinotecan-sensitive malignancies that have progressed on prior therapy (Onyx IST reference number: CAR-IST-553). Invest New Drugs.

[85] Zhu W, Liu J, Nie J (2015). MG132 enhances the radiosensitivity of lung cancer cells in vitro and in vivo. Oncol Rep.

[86] Kontopodis E, Kotsakis A, Kentepozidis N (2016). A phase II, open-label trial of bortezomib (VELCADE®) in combination with gemcitabine and cisplatin in patients with locally advanced or metastatic non-small cell lung cancer. Cancer Chemother Pharmacol.

[87] Voortman J, Smit EF, Honeywell R (2007). A parallel dose-escalation study of weekly and twice-weekly bortezomib in combination with gemcitabine and cisplatin in the first-line treatment of patients with advanced solid tumors. Clin Cancer Res.

[88] Baker AF, Hanke NT, Sands BJ (2014). Carfilzomib demonstrates broad anti-tumor activity in pre-clinical non-small cell and small cell lung cancer models. J Exp Clin Cancer Res.

[89] Ceresa C, Giovannetti E, Voortman J (2009). Bortezomib induces schedule-dependent modulation of gemcitabine pharmacokinetics and pharmacodynamics in non-small cell lung cancer and blood mononuclear cells. Mol Cancer Ther.

[90] de Wilt LHAM, Kroon J, Jansen G (2013). Bortezomib and TRAIL: a perfect match for apoptotic elimination of tumour cells?. Crit Rev Oncol Hematol.

[91] Cron KR, Zhu K, Kushwaha DS (2013). Proteasome inhibitors block DNA repair and radiosensitize non-small cell lung cancer. PLoS One.

[92] Mortenson MM, Schlieman MG, Virudachalam S (2005). Reduction in BCL-2 levels by 26S proteasome inhibition with bortezomib is associated with induction of apoptosis in small cell lung cancer. Lung Cancer.

[93] Shen J, Song G, An M (2014). The use of hollow mesoporous silica nanospheres to encapsulate bortezomib and improve efficacy for non-small cell lung cancer therapy. Biomaterials.

[94] Ao L, Reichel D, Hu D (2015). Polymer micelle formulations of proteasome inhibitor farfilzomib for improved metabolic stability and anticancer efficacy in human multiple myeloma and ung cancer cell lines. J Pharmacol Exp Ther.

[95] Li C, Hu J, Li W (2017). Combined bortezomib-based chemotherapy and p53 gene therapy using hollow mesoporous silica nanospheres for p53 mutant non-small cell lung cancer treatment. Biomater Sci.

[96] Huang C, Yokomise H, Miyatake A (2007). Clinical significance of the p53 pathway and associated gene therapy in non-small cell lung cancers. Future Oncol.

[97] Neukirchen J, Meier A, Rohrbeck A (2007). The proteasome inhibitor bortezomib acts differently in combination with p53 gene transfer or cytotoxic chemotherapy on NSCLC cells. Cancer Gene Ther.

[98] Nawrocki ST, Carew JS, Dunner K (2005). Bortezomib Inhibits PKR-Like Endoplasmic Reticulum (ER) Kinase and Induces Apoptosis via ER Stress in Human Pancreatic Cancer Cells. Cancer Res.

[99] Chiu HW, Lin SW, Lin LC (2015). Synergistic antitumor effects of radiation and proteasome inhibitor treatment in pancreatic cancer through the induction of autophagy and the downregulation of TRAF6. Cancer Lett.

[100] Fang J, Rhyasen G, Bolanos L (2012). Cytotoxic effects of bortezomib in myelodysplastic syndrome/acute myeloid leukemia depend on autophagy-mediated lysosomal degradation of TRAF6 and repression of PSMA1. Blood.

[101] Min H, Xu M, Chen ZR (2014). Bortezomib induces protective autophagy through AMP-activated protein kinase activation in cultured pancreatic and colorectal cancer cells. Cancer Chemother Pharmacol.

[102] Kroemer G, Levine B (2008). Autophagic cell death: the story of a misnomer. Nat Rev Mol Cell Biol.

[103] Yu L, Wan F, Dutta S (2006). Autophagic programmed cell death by selective catalase degradation. Proc Natl Acad Sci USA.

[104] Naumann K, Schmich K, Jaeger C (2012). Noxa/Mcl-1 balance influences the effect of the proteasome inhibitor MG-132 in combination with anticancer agents in pancreatic cancer cell lines. Anticancer Drugs.

[105] Gong L, Yang B, Xu M (2014). Bortezomib-induced apoptosis in cultured pancreatic cancer cells is associated with ceramide production. Cancer Chemother Pharmacol.

[106] Millward M, Price T, Townsend A (2012). Phase 1 clinical trial of the novel proteasome inhibitor marizomib with the histone deacetylase inhibitor vorinostat in patients with melanoma, pancreatic and lung cancer based on in vitro assessments of the combination. Invest New Drugs.

[107] Coelho SC, Almeida GM, Santos-Silva F (2016). Enhancing the efficiency of bortezomib conjugated to pegylated gold nanoparticles: an in vitro study on human pancreatic cancer cells and adenocarcinoma human lung alveolar basal epithelial cells. Expert Opin Drug Deliv.

[108] Tseng LM, Liu CY, Chang KC (2012). CIP2A is a target of bortezomib in human triple negative breast cancer cells. Breast Cancer Res.

[109] Chen YJ, Yeh MH, Yu MC (2013). Lapatinib-induced NF-kappaB activation sensitizes triple-negative breast cancer cells to proteasome inhibitors. Breast Cancer Res.

[110] Chang H, Huang T, Chen N (2014). Combination therapy targeting ectopic ATP synthase and 26S proteasome induces ER stress in breast cancer cells. Cell Death Dis.

[111] Gu Y, Bouwman P, Greco D (2014). Suppression of BRCA1 sensitizes cells to proteasome inhibitors. Cell Death Dis.

[112] Komatsu S, Miyazawa K, Moriya S et al (2012) Clarithromycin enhances bortezomib-induced cytotoxicity via endoplasmic reticulum stress-mediated CHOP (GADD153) induction and autophagy in breast cancer cells. Int J Oncol 1029–1039. 10.3892/ijo.2011.1317 10.3892/ijo.2011.1317 PMC3584821 22200786

[113] Miyahara K, Kazama H, Kokuba H (2016). Targeting bortezomib-induced aggresome formation using vinorelbine enhances the cytotoxic effect along with ER stress loading in breast cancer cell lines. Int J Oncol.

[114] Shi Y, Yu Y, Wang Z (2016). Second-generation proteasome inhibitor carfilzomib enhances doxorubicin-induced cytotoxicity and apoptosis in breast cancer cells. Oncotarget.

[115] Wang H, Yu Y, Jiang Z (2016). Next-generation proteasome inhibitor MLN9708 sensitizes breast cancer cells to doxorubicin-induced apoptosis. Sci Rep.

[116] Maynadier M, Basile I, Gallud A (2016). Combination treatment with proteasome inhibitors and antiestrogens has a synergistic effect mediated by p21WAF1 in estrogen receptor-positive breast cancer. Oncol Rep.

[117] Lin Y-C, Chen K-C, Chen C-C (2012). CIP2A-mediated Akt activation plays a role in bortezomib-induced apoptosis in head and neck squamous cell carcinoma cells. Oral Oncol.

[118] Li C, Johnson DE (2013). Liberation of functional p53 by proteasome inhibition in human papilloma virus-positive head and neck squamous cell carcinoma cells promotes apoptosis and cell cycle arrest. Cell Cycle.

[119] Zang Y, Thomas SM, Chan ET (2012). Carfilzomib and ONX 0912 inhibit cell survival and tumor growth of head and neck cancer and their activities are enhanced by suppression of Mcl-1 or autophagy. Clin Cancer Res.

[120] Gilbert J, Lee JW, Argiris A (2012). Phase II 2-arm trial of the proteasome inhibitor, PS-341 (bortezomib) in combination with irinotecan or PS-341 alone followed by the addition of irinotecan at time of progression in patients with locally recurrent or metastatic squamous cell carcinoma. Head Neck.

[121] Sung ES, Park KJ, Choi HJ (2012). The proteasome inhibitor MG132 potentiates TRAIL receptor agonist-induced apoptosis by stabilizing tBid and Bik in human head and neck squamous cell carcinoma cells. Exp Cell Res.

[122] Chang I, Wang CY (2016). Inhibition of HDAC6 protein enhances bortezomib-induced apoptosis in Head and Neck Squamous Cell Carcinoma (HNSCC) by reducing autophagy. J Biol Chem.

[123] Yim JH, Yun HS, Lee SJ (2016). Radiosensitizing effect of PSMC5, a 19S proteasome ATPase, in H460 lung cancer cells. Biochem Biophys Res Commun.

[124] Mehta A, Zhang L, Boufraqech M (2015). Carfilzomib is an effective anticancer agent in anaplastic thyroid cancer. Endocr Relat Cancer.

[125] Altmann A, Markert A, Askoxylakis V (2012). Antitumor effects of proteasome inhibition in anaplastic thyroid carcinoma. J Nucl Med.

[126] Zhang L, Boufraqech M, Lake R, Kebebew E (2016). Carfilzomib potentiates CUDC-101-induced apoptosis in anaplastic thyroid cancer. Oncotarget.

[127] Qiang W, Sui F, Ma J (2017). Proteasome inhibitor MG132 induces thyroid cancer cell apoptosis by modulating the activity of transcription factor FOXO3a. Endocrine.

[128] Zhang HY, Du ZX, Meng X (2013). Beclin 1 enhances proteasome inhibition-mediated cytotoxicity of thyroid cancer cells in macroautophagy-independent manner. J Clin Endocrinol Metab.

[129] Chen YJ, Wu H, Shen XZ (2016). The ubiquitin-proteasome system and its potential application in hepatocellular carcinoma therapy. Cancer Lett.

[130] Vandewynckel Y, Coucke C, Laukens D (2016). Next-generation proteasome inhibitor oprozomib synergizes with modulators of the unfolded protein response to suppress hepatocellular carcinoma. Oncotarget.

[131] Baiz D, Dapas B, Farra R (2014). Bortezomib effect on E2F and cyclin family members in human hepatocellular carcinoma cell lines. World J Gastroenterol.

[132] Witort E, Lulli M, Carloni V, Capaccioli S (2013). Anticancer activity of an antisense oligonucleotide targeting TRADD combined with proteasome inhibitors in chemoresistant hepatocellular carcinoma cells. J Chemother.

[133] Chen H, Yang H, Pan L (2016). The molecular mechanisms of XBP-1 gene silencing on IRE1α-TRAF2-ASK1-JNK pathways in oral squamous cell carcinoma under endoplasmic reticulum stress. Biomed Pharmacother.

[134] Liu D, Gao M, Yang Y (2015). Inhibition of autophagy promotes cell apoptosis induced by the proteasome inhibitor MG-132 in human esophageal squamous cell carcinoma EC9706 cells. Oncol Lett.

[135] Dang L, Wen F, Yang Y (2014). Proteasome inhibitor MG132 inhibits the proliferation and promotes the cisplatin-induced apoptosis of human esophageal squamous cell carcinoma cells. Int J Mol Med.

[136] Yang X (2012). Proteasome inhibitor bortezomi-induced the apoptosis of laryngeal squamous cell carcinoma Hep-2 cell line via disrupting redox equilibrium. Biomed Pharmacother.

[137] Li D, Lu Y, Sun P (2015). Inhibition on proteasome B1 subunit might contribute to the anti-cancer effects of fangchinoline in human prostate cancer cells. PLoS One.

[138] Modernelli A, Naponelli V, Giovanna Troglio M (2015). EGCG antagonizes Bortezomib cytotoxicity in prostate cancer cells by an autophagic mechanism. Sci Rep.

[139] Chattopadhyay N, Berger AJ, Koenig E (2015). KRAS genotype correlates with proteasome inhibitor ixazomib activity in preclinical in vivo models of colon and non-small cell lung cancer: Potential role of tumor metabolism. PLoS One.

[140] Krȩtowski R, Borzym-Kluczyk M, Cechowska-Pasko M (2014). Efficient apoptosis and necrosis induction by proteasome inhibitor: bortezomib in the DLD-1 human colon cancer cell line. Mol Cell Biochem.

[141] Singha B, Gatla HR, Phyo S, Patel A (2015). IKK Inhibition Increases Bortezomib Effectiveness in Ovarian Cancer. Oncotarget.

[142] Denlinger CS, Meropol NJ, Li T (2014). A phase II trial of the proteasome inhibitor bortezomib in patients with advanced biliary tract cancers. Cancer Res.

[143] Ito K, Kobayashi M, Kuroki S (2013). The proteasome inhibitor bortezomib inhibits the growth of canine malignant melanoma cells in vitro and in vivo. Vet J.

[144] Selimovic D, Porzig BBOW, El-Khattouti A (2013). Bortezomib/proteasome inhibitor triggers both apoptosis and autophagy-dependent pathways in melanoma cells. Cell Signal.

[145] Franke NE, Niewerth D, Assaraf YG (2012). Impaired bortezomib binding to mutant β5 subunit of the proteasome is the underlying basis for bortezomib resistance in leukemia cells. Leukemia.

[146] Niewerth D, Kaspers GJL, Assaraf YG (2014). Interferon-γ-induced upregulation of immunoproteasome subunit assembly overcomes bortezomib resistance in human hematological cell lines. J Hematol Oncol.

[147] Niewerth D, van Meerloo J, Jansen G (2014). Anti-leukemic activity and mechanisms underlying resistance to the novel immunoproteasome inhibitor PR-924. Biochem Pharmacol.

[148] Suzuki E, Demo S, Deu E (2011). Molecular mechanisms of bortezomib resistant adenocarcinoma cells. PLoS One.

[149] Weyburne ES, Wilkins OM, Sha Z (2017). Inhibition of the proteasome β2 site sensitizes triple-negative breast cancer cells to β5 inhibitors and suppresses Nrf1 activation. Cell Chem Biol.

[150] Niewerth D, Franke NE, Jansen G (2013). Higher ratio immune versus constitutive proteasome level as novel indicator of sensitivity of pediatric acute leukemia cells to proteasome inhibitors. Haematologica.

[151] Nguyen M, Marcellus RC, Roulston A (2007). Small molecule obatoclax (GX15–070) antagonizes MCL-1 and overcomes MCL-1-mediated resistance to apoptosis. Proc Natl Acad Sci.

[152] Miyamoto Y, Hosotani R, Wada M (1999). Immunohistochemical analysis of Bcl-2, Bax, Bcl-X, and Mcl-1 expression in pancreatic cancers. Oncology.

[153] Zang Y, Kirk CJ, Johnson DE (2014). Carfilzomib and oprozomib synergize with histone deacetylase inhibitors in head and neck squamous cell carcinoma models of acquired resistance to proteasome inhibitors. Cancer Biol Ther.

[154] Ao L, Wu Y, Kim D (2012). Development of peptide-based reversing agents for P-glycoprotein-mediated resistance to carfilzomib. Mol Pharm.

[155] Verbrugge SE, Assaraf YG, Dijkmans BA (2012). Inactivating PSMB5 mutations and P-glycoprotein (multidrug resistance-associated protein/ATP-binding cassette B1) mediate resistance to proteasome inhibitors: ex vivo efficacy of (immuno)proteasome inhibitors in mononuclear blood cells from patients with. J Pharmacol Exp Ther.

[156] Oerlemans R, Franke NE, Assaraf YG (2008). Molecular basis of bortezomib resistance: proteasome subunit beta5 (PSMB5) gene mutation and overexpression of PSMB5 protein. Blood.

[157] de Bruin G, Xin BT, Kraus M (2016). A set of activity-based probes to visualize human (immuno) proteasome activities. Angew Chem Int Ed Engl.

[158] Lee SJ, Levitsky K, Parlati F (2016). Clinical activity of carfilzomib correlates with inhibition of multiple proteasome subunits : application of a novel pharmacodynamic assay. Br J Cancer.

[159] Seifert U, Bialy LP, Ebstein F (2010). Immunoproteasomes preserve protein homeostasis upon interferon-induced oxidative stress. Cell.

[160] Yun YS, Kim KH, Tschida B (2016). mTORC1 coordinates protein synthesis and immunoproteasome formation via PRAS40 to prevent accumulation of protein stress. Mol Cell.

[161] Muchamuel T, Basler M, Aujay MA (2009). A selective inhibitor of the immunoproteasome subunit LMP7 blocks cytokine production and attenuates progression of experimental arthritis. Nat Med.

[162] Raz S, Sheban D, Gonen N (2014). Severe hypoxia induces complete antifolate resistance in carcinoma cells due to cell cycle arrest. Cell Death Dis.

[163] Franke NE, Kaspers GL, Assaraf YG (2016). Exocytosis of polyubiquitinated proteins in bortezomib-resistant leukemia cells: a role for MARCKS in acquired resistance to proteasome inhibitors. Oncotarget.

[164] Green TD, Crews AL, Park J (2011). Regulation of mucin secretion and inflammation in asthma: a role for MARCKS protein?. Biochim Biophys Acta.

[165] Chen CH, Thai P, Yoneda K (2014). A peptide that inhibits function of Myristoylated Alanine-Rich C Kinase Substrate (MARCKS) reduces lung cancer metastasis. Oncogene.

[166] Micallef J, Dharsee M, Chen J (2010). Applying mass spectrometry based proteomic technology to advance the understanding of multiple myeloma. J Hematol Oncol.

[167] Zaal EA, Wu W, Jansen G (2017). Bortezomib resistance in multiple myeloma is associated with increased serine synthesis. Cancer Metab.

[168] Li X, Wood TE, Sprangers R (2010). Effect of noncompetitive proteasome inhibition on bortezomib resistance. J Natl Cancer Inst.

[169] Arcy PD, Brnjic S, Olofsson MH (2011). Inhibition of proteasome deubiquitinating activity as a new cancer therapy. Nat Med.

